# Computer-Assisted Navigation During an Anterior-Posterior En Bloc Resection of a Sacral Tumor

**DOI:** 10.7759/cureus.373

**Published:** 2015-11-04

**Authors:** Sami Al Eissa, Amro F Al-Habib, Faisal R Jahangiri

**Affiliations:** 1 Division of Orthopedics, Department of Surgery, King Abdulaziz Medical City, Ministry of National Guard Health Affairs, Riyadh, Saudi Arabia; 2 Neurosurgery Division, Department of Surgery, King Khalid University Hospital, College of Medicine, King Saud University, Riyadh, Saudi Arabia; 3 Division of Neurology, Department of Medicine, King Abdulaziz Medical City, Ministry of National Guard Health Affairs, Riyadh, Saudi Arabia

**Keywords:** sacrectomy, en bloc, ewing’s sarcoma, intraoperative neurophysiological montoring, computer-assisted navigation system

## Abstract

Previously, a computer-based navigation system has not been used routinely for en-bloc resection of sacral tumors. In order to improve the accuracy of tumor resection, O-arm navigation was used to join anterior and posterior osteotomies during an en-bloc resection of a sacral Ewing’s sarcoma.

This case study describes the technique for en-bloc resection of a sacral Ewing’s sarcoma guided by O-arm computer navigation and intraoperative neurophysiological monitoring (IONM).

An 18-year-old male presented with weakness in his left lower extremity. MRI of the patient's spine showed a sacral mass causing compression of left S1 and S2 roots. A surgical resection was planned with anterior and posterior approaches. An O-arm computer navigation system was used to assist in meeting anterior osteotomy cuts with the posterior cuts to ensure complete resection of the sacral tumor with a safe margin.

Computer-assisted navigation was used along with IONM during this procedure to help guide the surgical team in an adequate tumor resection. There were no complications related to the use of the O-arm or the navigation system.

Computer navigation guidance is both useful and safe in sacral tumor resections. It enhanced the accuracy of the en-bloc removal of a sacral tumor with safe margins while protecting neural function and minimizing recurrence.

## Introduction

A computer-assisted navigation system is a computer-assisted, image-based, surgical guidance through the bone anatomy. The computer coordinates images from O-arm scans of the patient and a camera attached to the instrument in use. It is a system that enables surgeons to visualize the anatomy of a patient’s spine during surgery and to precisely track the location of their surgical instruments in relation to the anatomy. With the use of computer-assisted navigation, surgeons are able to to perform less invasive procedures, reduce radiation exposure for the surgeon and staff, and help in improving the patient’s clinical outcome [1-2]. The most common use of the navigation system is in spine surgery for pedicle screw insertion, especially in complicated cases [3]. En bloc resection of sacral chordomas is difficult, but now the surgeon can frequently perform en bloc resections of a sacral tumor with safe margins with newly developed surgical and imaging techniques [4]. The main objective of our case management was to precisely join anterior and posterior cuts on the sacrum and remove the tumor with safe margins.

## Case presentation

### Clinical assessment and imaging

An 18-year-old male patient presented with minimal weakness of the left ankle/toes plantar-flexion, which worsened with gait.

**Figure 1:**
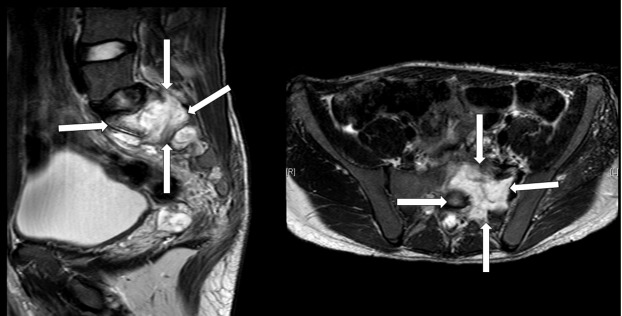
A preoperative T2-weighted MRI scan showing a tumor mass invading the sacral bone (white arrows).

The magnetic resonance imaging (MRI) showed a large sacral mass causing compression of the S1 and S2 roots on the left side. It was hypointense in T1 and hyperintense in T2, originating mainly from the left side of S1, extending into the left side of S2 with extension into the left paravertebral space and through the neural foramen into the extradural intraspinal canal, causing severe compression and displacement of the thecal sac to the right side (Figure 1). It exerted a mass effect on the adjacent parts of the sigmoid, but there was no evidence of invasion. An invasion into the left iliac vein was noted. There was a suspected blood fluid level seen at the posterior aspect of the mass. The left sacroiliac joint was involved by the tumor. Informed patient consent was obtained for treatment.

**Figure 2:**
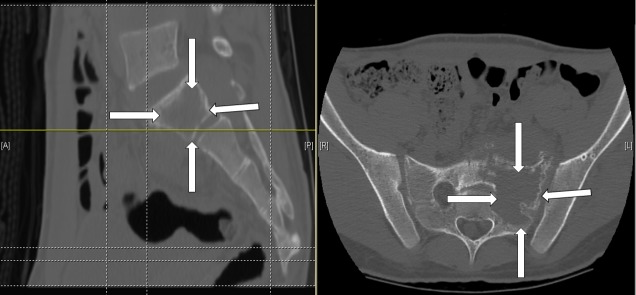
A preoperative CT scan showing the tumor mass invading the sacral bone (white arrows).

CT scan confirmed a large lytic lesion with a soft tissue mass at the left sacroiliac joint extending to the midline of the sacrum medially and to the sacroiliac joint laterally (Figure 2). No metastatic lesions were identified on other imaging.

Image-guided biopsy confirmed the diagnosis of Ewing’s sarcoma. Surgical resection of the tumor with safe margins was planned. Three sessions of chemotherapy were administered preoperatively to shrink the tumor.

### Surgery

**Figure 3:**
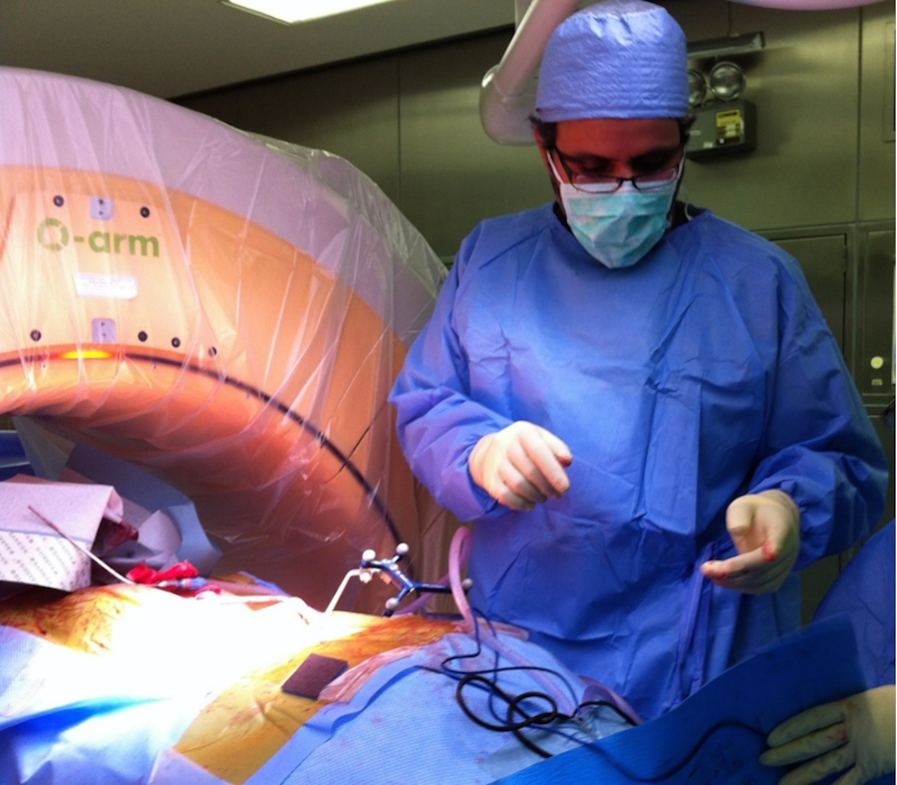
O-arm computer navigation system used in the operating room to combine anterior and posterior cuts.

A sacrectomy was planned as a two-stage procedure, anterior and posterior. During the anterior approach, the tumor was freed from major vessels and the bowel. Surface cuts were done in the iliac bone anteriorly with a 2 cm margin outside the tumor. These cuts were guided by the navigation system to be precisely outside the tumor (Figure 3). It served as markers on the navigation system when the posterior approach was performed as the second stage. During the posterior approach, the surgical procedure included lumbar spine instrumentation with right iliac screw placement, bilateral laminectomy of L5, S1-S2 and S3 to expose the dura with ligation of the left L5, S1, S2, and S3 roots. Navigator oriented posterior cortex cutting was performed around the mass precisely meeting the anterior cuts. The tumor was then removed in one piece. Reconstruction was performed using a fibular bone graft between the two iliac bones. The procedure was done under IONM for all four extremities (upper and lower). A multi-modality IONM was used including somatosensory evoked potentials (SSEP), transcranial electrical motor evoked potentials (TCeMEP), and electromyography (EMG). IONM was helpful during this surgery in guiding the surgical team and in preventing any damage to sacral roots on the normal right side and preventing any positional neurapraxia in upper extremities [5-6].

### Postoperative care

The patient recovered well from surgery. He was discharged from the hospital with no complications. Pathology confirmed Ewing’s sarcoma. A post-surgical MRI showed complete tumor removal with sufficient resection margins. Sphincter function was normal with left L5 weakness one week postoperatively. Another three sessions of chemotherapy were started soon after surgery; radiotherapy was delayed about six weeks. A follow-up examination of the patient at one, two, and three years showed no signs of reoccurrence.

## Discussion

In only 3-10% of Ewing’s sarcomas, the principal site is the spine. The extra-spinal site for a metastatic lesion is usually common. In children and between ages of 10-30 years the Ewing’s sarcoma is the most common primary non-lymphoproliferative malignant tumor. The most common site for these tumors is the lumbosacral spine, usually in spinal vertebral bodies. CT scan and MRI typically show paraspinal soft tissue involvement including extradural spaces. The lesions are commonly seen as lytic, mixed or sclerotic on imaging.

**Figure 4:**
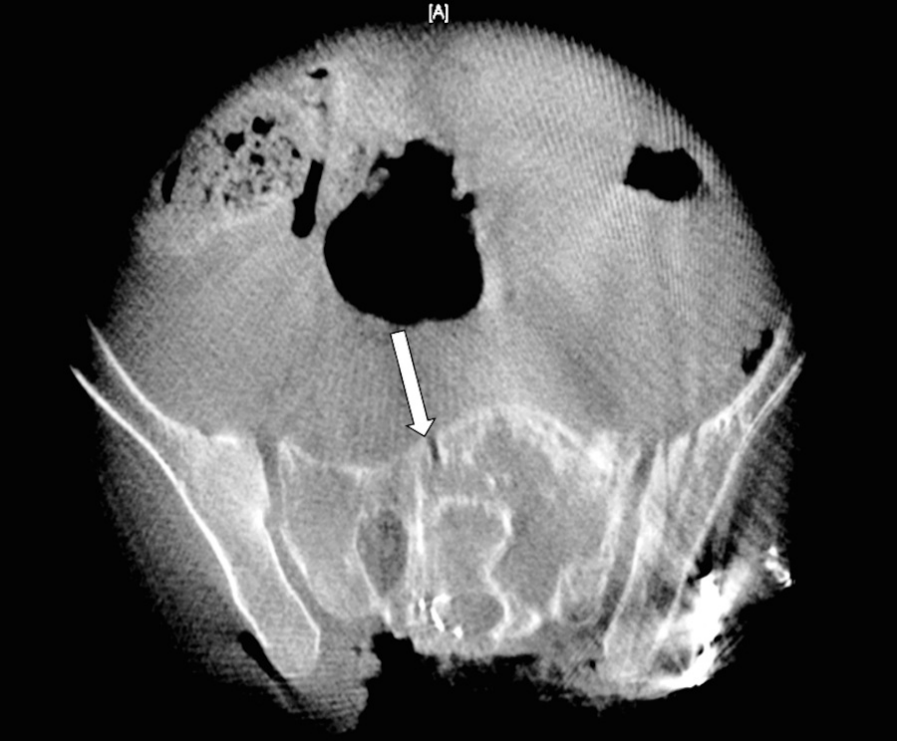
Intra-operative O-arm image showing the anterior cut placed on the sacrum (white arrow) during Stage 1 of the procedure (anterior approach).

The first stage was an anterior approach, lasting nine hours. After releasing the anterior structures attached to the tumor, an anterior cut was made in the sacrum to the right side of the tumor with a safe margin (Figure 4).

**Figure 5:**
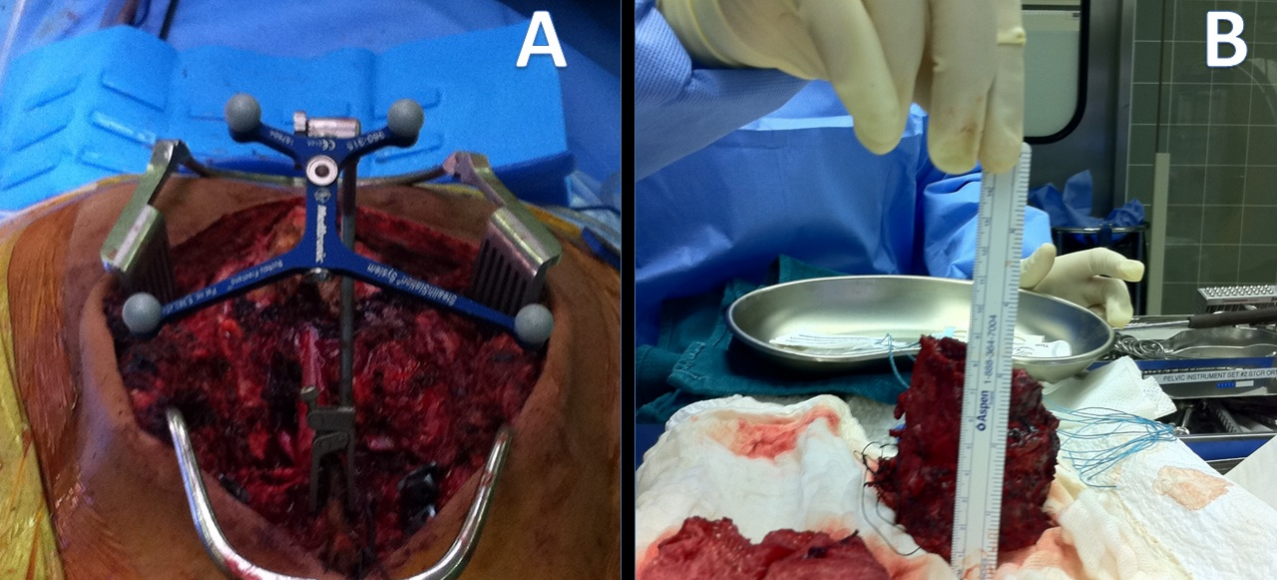
Post-tumor resection. A) Intra-operative navigation for identifying tumor anterior and posterior margins. B) En bloc tumor resected approximately 5.8 x 6 x 4 cm size.

The navigation system was used to make the cut precisely. The second 14 hours long stage was a posterior approach. After the anterior release and cut, the patient was flipped to a prone position. O-arm navigation system was used to identify the anterior cut, make an aligned posterior cut and then release the sacral tumor (Figure 5).

**Figure 6:**
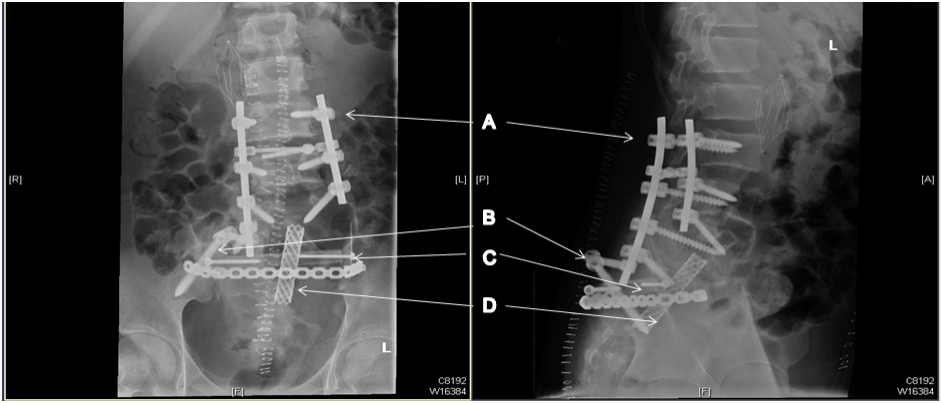
Postoperative MRI showing the post-resection and fusion spinal column. (A) Pedicle screw; (B) Iliac screw; (C) Fibular graft; (D) Strut.

**Figure 7:**
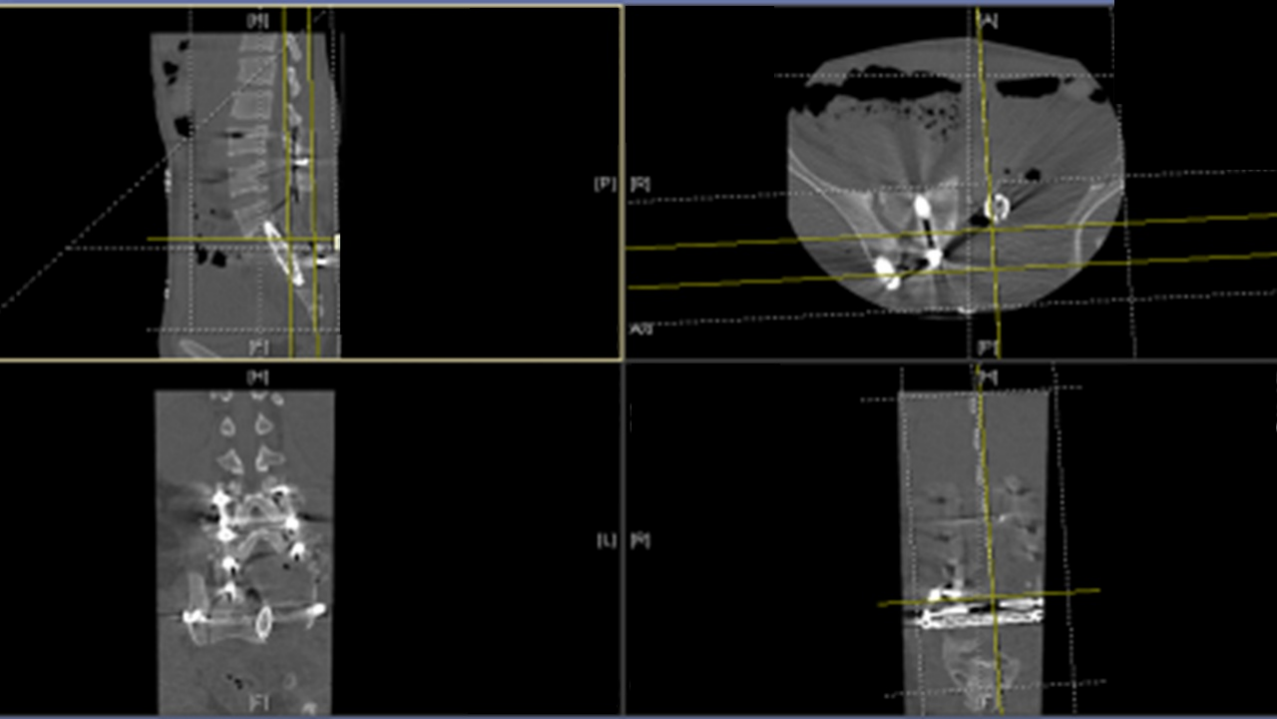
A postoperative CT showing the complex tumor resection with sufficient resection margins.

Pedicle screws, an iliac screw, strut and a fibular bone graft were used to stabilize the spine following the removal of the sacral bone structure (Figures 6-7). The surgical team included an ortho-spine surgeon, a neuro-spine surgeon, an ortho-onco surgeon, an ortho-pelvic surgeon and a vascular surgeon.

Computer-aided tumor resection was used previously to guide adequate tumor resection [7]. Jeys, et al. found that navigation guidance reduced the rate of intralesional resection in 23 primary malignant pelvic and sacral tumors [8]. Dosenbrock, et al. found the navigation-guided technology useful during en-bloc sacral chordoma resection [9]. It helped in making precise osteotomy cuts around tumor margins. This experience was shared in the current case. We also used the anterior cuts as landmarks for the margins anteriorly. These were met precisely during osteotomies from the posterior approach.

## Conclusions

Utilization of a computer-assisted navigation system during sacral tumor resection has not been routinely reported. This is a case report of computer-assisted en-bloc sacrectomy for an anterior and posterior tumor resection of a sacral tumor. Various studies have reported computer-guided navigation very useful during tumor resections. We decided to use the computer-guided navigation to make an anterior cut as a landmark margin. The anterior cut was met precisely by a posterior cut using computer-guided navigation. This technique helped us in making precise osteotomy cuts around the sacral tumor margins. We believe this anterior-posterior approach technique is superior to posterior-cut only and recommend using this technique for sacral tumor resections. The computer-guided navigation helps in making precise cuts around tumor by joining the anterior and posterior cuts under the navigation.
